# Method for evaluating the performance of catalytic reactions using renewable-energy-derived materials

**DOI:** 10.1038/s41598-022-14789-4

**Published:** 2022-06-22

**Authors:** Yuichi Manaka, Yuki Nagata, Keisuke Kobayashi, Daisuke Kobayashi, Tetsuya Nanba

**Affiliations:** 1 Renewable Energy Research Center, National Institute of Advanced Industrial Science and Technology, 2-2-9 Machiikedai, Koriyama, Fukushima 963-0298 Japan; 2 School of Material and Chemical Technology, Tokyo Institute of Technology, 2-12-1 Ookayama, Meguro-ku, Tokyo, 152-8552 Japan; 3 Department of Applied Chemistry, Tokyo Denki University, 5 Senju-Asahicho, Adachi-ku, Tokyo, 120-8551 Japan

**Keywords:** Catalysis, Hydrogen energy

## Abstract

Hydrogen produced by electrolysis using electricity derived from renewable energy sources has a fluctuating supply. However, conventional catalyst evaluation methods cannot evaluate catalyst synthesis assuming a fluctuating feedstock. This paper investigates a simple screening method for catalysts that can be used for renewable energy by using a combination of three catalyst evaluation methods: Light-off Performance, equilibrium achievement degree, and maximum ammonia concentration. We examined the combination of evaluation methods and trends for each element, and finally concluded that a three-axis graph combining the three is the easiest graph to obtain the information necessary for catalyst screening intuitively rather than quantitatively.

## Introduction

Storing generated electricity for use at an appropriate time and place (i.e., energy management) is a requirement for the effective use of renewable energy, which is a variable and decentralized energy source. For this purpose, a storage device, such as a battery or a flywheel, is required for short-term renewable-energy storage, while using electricity derived from renewable energy to electrolyze and chemically convert the energy into hydrogen is useful for large-scale handling or long-term storage^1^. However, handling hydrogen in its gaseous state is undesirable in terms of both the volumetric energy rate and safety. Therefore, hydrogenated chemicals, commonly referred to as “hydrogen carriers”, are proposed as hydrogen storage media. A variety of hydrogen carriers have been developed. Well-known hydrogen carriers include, for example, methane^2^, Liquid Organic Hydrogen Carrier^3^, methanol^4^, formic acid^5^, urea^5^, and molecules containing BN^6^. Among which ammonia (nitrogen hydride) is a promising molecule^7^. Ammonia can be decomposed to extract hydrogen for use^8^, or it can be used directly as fuel^9^. Although still in the research stage, there is a possibility of supplying ammonia in a variety of ways^10,11^. Ammonia has a hydrogen content of 17.6 wt% and is easily liquefied, which makes it easy to handle. In addition, since it is widely used as a raw material in modern chemical industries, significant infrastructure and handling know-how exist, which facilitates societal adaptation.

However, the synthesis of ammonia from hydrogen produced by water electrolysis from electricity obtained from renewable energy power generation presents a problem that has not been considered in catalytic chemistry or chemical engineering. In general chemical processes, the feedstock supply is always constant and the process is operated continuously for a long time. In contrast, feedstock derived from renewable energy sources (especially hydrogen in the case of ammonia synthesis) fluctuates in supply and energy, and depending on weather conditions, not only the feedstock supply but also the plant itself must be repeatedly started up and shut down. Synthesis plants that operate under such conditions do not exist^12^. Nor has there been any catalyst evaluation for such operations. This means that the catalyst needs to be evaluated for activity from low temperatures^13–16^.

However, it is not enough. The catalytic activity, which takes into account the reverse reaction, is another indicator to be evaluated. This is because the variability of the raw materials supplied from renewable energy sources also changes the temperature distribution in the catalyst bed, making the balance between production and decomposition extremely complex. Detailed kinetic analysis and reactor simulations may be able to address this issue, but a method to roughly assess the balance between formation and decomposition will be needed. The general evaluation of the highest catalytic activity is also an indicator that must be evaluated in the study of catalysts.

In this paper, we investigated a new method for evaluating catalysts for renewable energy applications. Ru/MgO-MOx catalysts with various metals added to MgO supports were prepared as catalysts for ammonia synthesis, and their catalytic activity for ammonia synthesis was investigated. This catalyst was considered appropriate for the evaluation method since it has such a reverse reaction activity that it can also be used for ammonia decomposition under some reaction conditions. Three catalyst characterization methods were used to evaluate the properties of each catalysts and its suitability for renewable-energy applications. Three methods are: low temperature activity, evaluation of the balance between production and decomposition, and maximum activity of the catalyst. A new catalyst evaluation method was studied by combining the results of the three evaluation methods, and finally a highly visible three-axis graph was developed to evaluate catalysts for renewable energy in a short period of time. The introduced evaluation method is considered useful for catalyst screening, etc., and will provide a new paradigm for the development of future synthetic reactions using renewable energy as a raw material or heat source.

## Results and discussion

MgO-MOx materials were prepared as catalysts for ammonia synthesis by adding various metal oxides to MgO supports, while Ru/MgO-MOx supports were prepared by adding Ru to the MgO-MOx supports^17,18^. Ammonia-synthesis activity was investigated by setting each catalyst in a fixed-bed reactor. The preparation of each catalyst and the method used to determine its catalytic activity are described in the “Methods” section. The obtained data were analyzed as detailed below.

### Light-off performance

**Figure 1:**
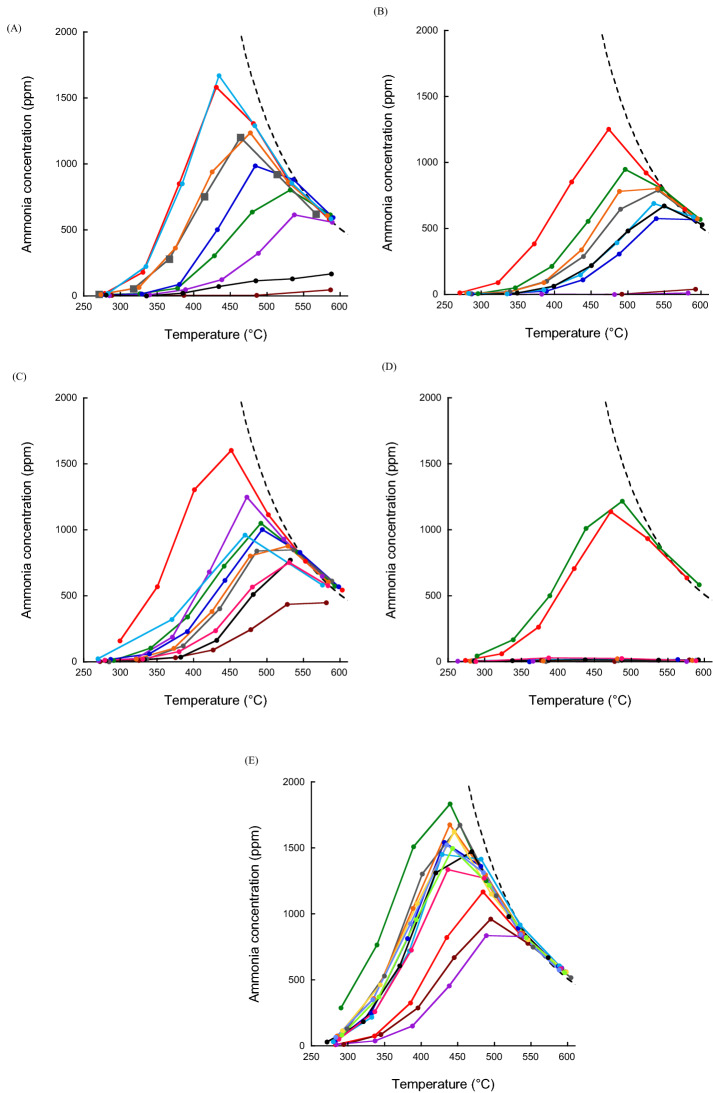
Concentrations of NH_3_ synthesized using various Ru/MgO-MO_x_ catalyst as functions of temperature. (**A**) The addition of alkali metals and alkaline earth metals. The black dotted line corresponds to the equilibrium conversion. Color code: red line: lithium, green line: sodium, blue line: potassium, brown line: rubidium, purple line: cesium, gray squares: magnesium, orange line: calcium, light blue line: strontium, black line: barium. (**B**) The addition of transition metals (period 4). The black dotted line corresponds to the equilibrium conversion. Color code: red line: scandium, green line: titanium, blue line: vanadium, brown line: chromium, purple line: manganese, gray line: iron, orange line: cobalt, light blue line: nickel, black line: copper. (**C**) The addition of transition metals (period 5 and 6 without lanthanides). The black dotted line corresponds to the equilibrium conversion. Color code: red line: yttrium, green line: zirconium, blue line: niobium, brown line: molybdenum, purple line: rhodium, gray line: palladium, orange line: silver, light blue line: rhenium, black line: iridium, pink line: platinum. (**D**) The addition of Lanthanides. The black dotted line corresponds to the equilibrium conversion. Color code: red line: lanthanum, green line: cerium, blue line: praseodymium, brown line: neodymium, purple line: samarium, gray line: europium, orange line: gadolinium, light blue line: terbium, black line: dysprosium, pink line: holmium, yellow line: erbium, light green: thulium, pale blue line: ytterbium. (**E**) The addition of p-block elements and zinc. The black dotted line corresponds to the equilibrium conversion. Color code: red line: aluminum, green line: silicon, blue line: phosphorus, brown line: sulfur, purple line: zinc, gray line: gallium, orange line: indium, light blue line: tin, black line: tellurium, pink line: bismuth. Reaction conditions are described in the “Methods” section.

The light-off performance of each catalyst was first evaluated; the evaluation method used herein is often used to evaluate automotive catalysts^19,20^, as an on-board exhaust gas-reduction catalyst needs to be catalytically active when the engine is turned on from a stationary low-temperature state. Renewable energy-enabled plants may start up frequently and hydrogen derived from renewable energy sources may be supplied at low temperatures^21^; hence, evaluating the temperature at which the catalyst becomes catalytically active is important. The ammonia synthesis results obtained using the various catalyst are shown in Fig. 1, which shows that the catalysts exhibit higher activities when lanthanides are included; such observations have been reported in previous studies^22^. The addition of an alkali metal is well-known to improve catalytic activity^23^; however, the effect was not significant in this study because alkali metals are included as oxides in the support rather than as an added third component. With the exception of Al and Si, the addition of a p-block element led to lower activity than the catalyst on the MgO support.

The rate constant, *k*, for ammonia synthesis was calculated from the measured ammonia concentration [NH_3_] (volppm) obtained at each experimental temperature (Fig. 1) using the following Eq. (1):

1 $$k\left( {{\text{mol min}}^{{ - {1}}} {\text{g}}^{{ - {1}}} } \right) \, = \, \left[ {{\text{NH}}_{{3}} } \right] \times {8}0/({1,}000,000 \times {22,4}00 \times 0.{2})$$

The natural logarithm of the ammonia synthesis rate constant *k* (ln(*k*)) between 260 and 430 °C for each catalyst was plotted as a function of reciprocal reaction temperature (1000/K). The “light-off value” is the reciprocal temperature at which 50 ppm of ammonia is produced, is obtained by linear regression extrapolation, and is an indicator of the lowest temperature at which the catalyst exhibits high activity. In other words, it corresponds to the temperature that enables the catalytic reaction to start well. The reciprocal numbers are used for visibility in this study, and a graph with the axes reversed is also acceptable.

When relying on materials derived from renewable energy sources to drive catalytic reactions, the possibility of intermittent supply or the lack of supply for some time is a real possibility. The ability to start well should be considered as a criterion when evaluating catalysts. In addition, mathematical extrapolation led to negative light-off values for some catalysts. Catalysts with negative values have very low catalytic activities (Fig. 1) and are strongly affected by measurement errors, which explains the apparent negative values. Combinations of the light-off value and other indices are discussed in the following sections.

### Achieving equilibrium

The second method used to evaluate catalysts for renewable energy applications is the degree to which equilibrium is achieved, which is a measure of the catalytic activities on the balance between formation and decomposition of ammonia to reach the thermodynamic equilibrium concentration of ammonia. Low-temperature and high-pressure conditions are well-known to be preferable for the synthesis of ammonia, as high temperatures lead to low equilibrium concentrations. A higher temperature generally leads to a concentration of generated ammonia that approaches the equilibrium concentration (Fig. 1). A high equilibrium achievement means that the result of the catalytic reaction is close to the chemical equilibrium value, which means that the catalyst is not much affected by the reverse reaction. Reaction order analysis is often used to evaluate the effect of the reverse reaction. This kinetic evaluation method has been used in many research, and is particularly important for the evaluation of activity at low temperatures. However, the order of reaction is not necessarily the dominant factor in the evaluation of maximum catalytic activity or in the evaluation of the effect of the reverse reaction at higher temperatures. For example, it has been shown kinetically that the ammonia produced at high temperatures contributes to the reduction of hydrogen inhibition^24^. The equilibrium degree, which is a thermodynamic value, was used to lump together the factors involved in the various reactions at high temperatures into a simple index.

**Figure 2:**
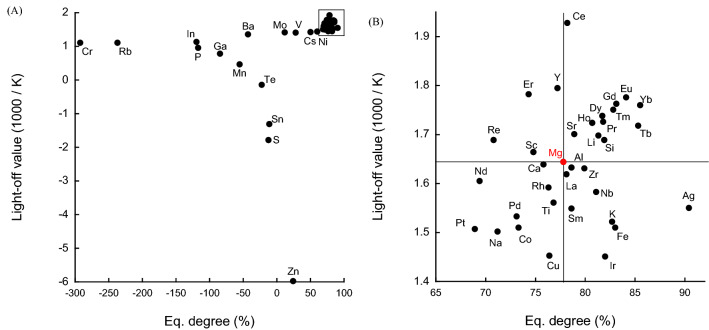
Correlation between Light-off value and equilibrium achievement degree. (**A**) All catalysts. (**B**) Expanded view of the enclosed area in panel (**A**). The elemental symbols correspond to the added elements.

The slope (*S*) was calculated by taking the reciprocal of the reaction temperature (1000/K) on the X-axis and the natural logarithm of the ammonia synthesis rate constant *k* (ln(*k*)) on the Y-axis in the range of 450–600 °C. From the thermodynamic equilibrium concentration of ammonia at each temperature, the slope *(S*_e_) was determined to be in the range of 300–600 °C. *S*/*S*_e_ × 100, which is referred to as the “equilibrium achievement degree” (Eq. degree) was calculated as a measure of the closeness to equilibrium of the catalyst. Figure 2 shows two-dimensional catalyst evaluation plots of light-off value as a function of equilibrium degree.

As mentioned above, the method used to calculate the light-off value sometimes led to negative values for materials with very low catalytic activities that barely produce ammonia at high temperatures (Fig. 2A). In this regard, further studies into the calculation method may be required. Catalysts with lower activities tended to have lower light-off values and lower equilibrium achievement degrees. Catalysts with some activity were plotted in different locations for each catalyst (Fig. 2, right). Because the support was prepared by adding various metals to MgO, the result for Mg alone, highlighted in red in Fig. 2, is the criterion for determining catalytic activity. Catalyst additives located in the positive X-direction from the Mg value have a positive effect on catalytic activity, while those located in the negative direction affect catalytic activity negatively; the same trend is observed for the Y-axis, where catalyst additives located in the positive direction of the Mg point have a positive effect on catalytic activity, while those located in the negative direction have a negative effect. The catalysts located in the upper left corner of the figure have lower activation temperatures and deviate from the equilibrium concentration. In other words, further catalyst improvement will bring them closer to equilibrium. On the other hand, the plot position may also indicate catalyst degradation at high temperatures, which reflects high-temperature durability and changes in the catalytic reaction pathway due to surface changes at high temperatures. The catalyst in the lower left corner of the figure contains metals that easily form complexes with ammonia such as Co, Cu and Pt. There may be a special interaction with ammonia before approaching the equilibrium concentration. The catalyst located in the lower right corner of the figure is activated at a higher temperature; however, ammonia is produced at a concentration close to the equilibrium concentration after activation. This catalyst is expected to be used in the second stage of a multi-step process, as it exhibits a low reverse-reaction effect. The lanthanide-doped catalysts are mostly plotted in the upper right of the figure. In addition to the simple improvement of low temperature activity, the lanthanide-doped catalysts may have the property that the reaction pathway does not change too complicatedly even at high temperatures. Determining the equilibrium achievement value provides a rough understanding of the properties of a catalyst without the need to accurately determine the order of ammonia by detailed kinetic analysis. Ru/MgO catalysts are also capable of decomposing ammonia under certain reaction conditions^25–27^. Catalysts with more-negative equilibrium achievement values than Mg are possibly useful as catalysts for the production of hydrogen by the decomposition of ammonia.

### Maximum ammonia concentration

As the third method for evaluating catalysts for renewable energy applications, we calculated the expected maximum ammonia concentration produced by the catalyst, which involves interpolating between temperatures over the entire experimental temperature range. The calculated maximum ammonia concentration is defined as the maximum amount synthesizable by the catalyst, is an indicator of catalyst activity, and is generally used to evaluate all catalysts, not just those intended for renewable energy applications.

**Figure 3:**
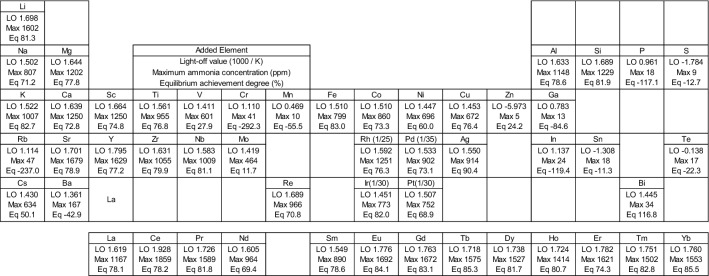
Light-off values (1000/K), equilibrium achievement degrees (%), and maximum ammonia concentrations (ppm) for the catalysts in this study according to the added element.

The light-off values, equilibrium achievement degrees, and maximum ammonia concentrations for each catalyst are displayed in Fig. 3 in periodic table form. It was difficult to determine which are good catalysts based on intra-group or periodic trends.

**Figure 4:**
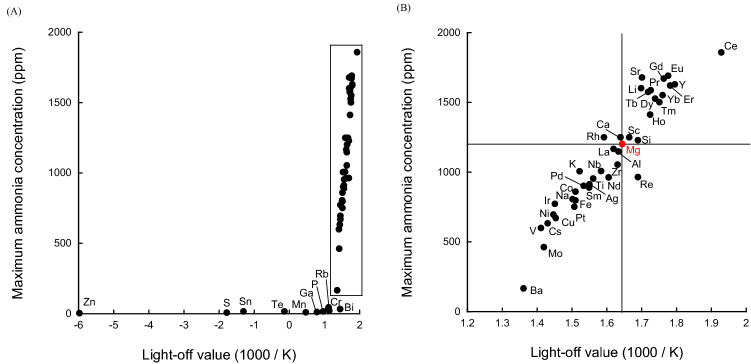
Correlation between maximum ammonia concentration and light-off value. (**A**) All catalysts. (**B**) Enlarged view of the enclosed area in panel (**A**). The elemental symbols correspond to the added elements.

Consequently, we examined the relationship between maximum ammonia concentration and light-off value (Fig. 4).

A linear relationship was observed between the light-off value and the maximum ammonia concentration at light-off values above unity. Catalysts with higher low-temperature activities exhibited higher maximum ammonia concentrations. The effect of the added metal can be determined in a similar manner to that in Fig. 2, using the Mg as the reference point.

**Figure 5:**
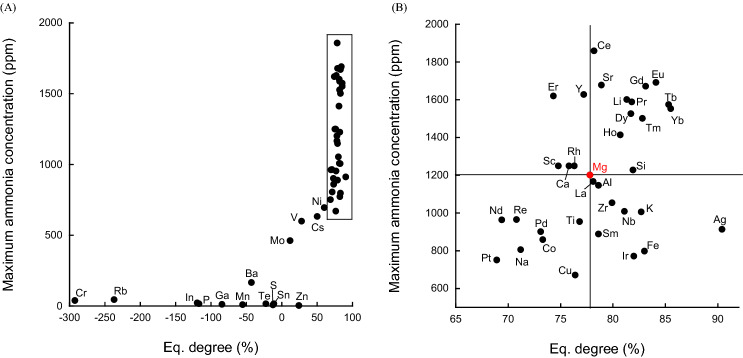
Correlation between maximum ammonia concentration and equilibrium achievement degree. (**A**) All catalysts. (**B**) Enlarged view of the enclosed area in panel (**A**). The elemental symbols correspond to the added elements.

Figure 5 shows the relationship between equilibrium achievement degree and maximum ammonia temperature. As Fig. 4 shows that a linear relationship exists between the light-off value and the maximum ammonia concentration, the relationship between the equilibrium achievement degree and the maximum ammonia concentration in Fig. 5 shows the same degree of dispersion as Fig. 4.

**Figure 6:**
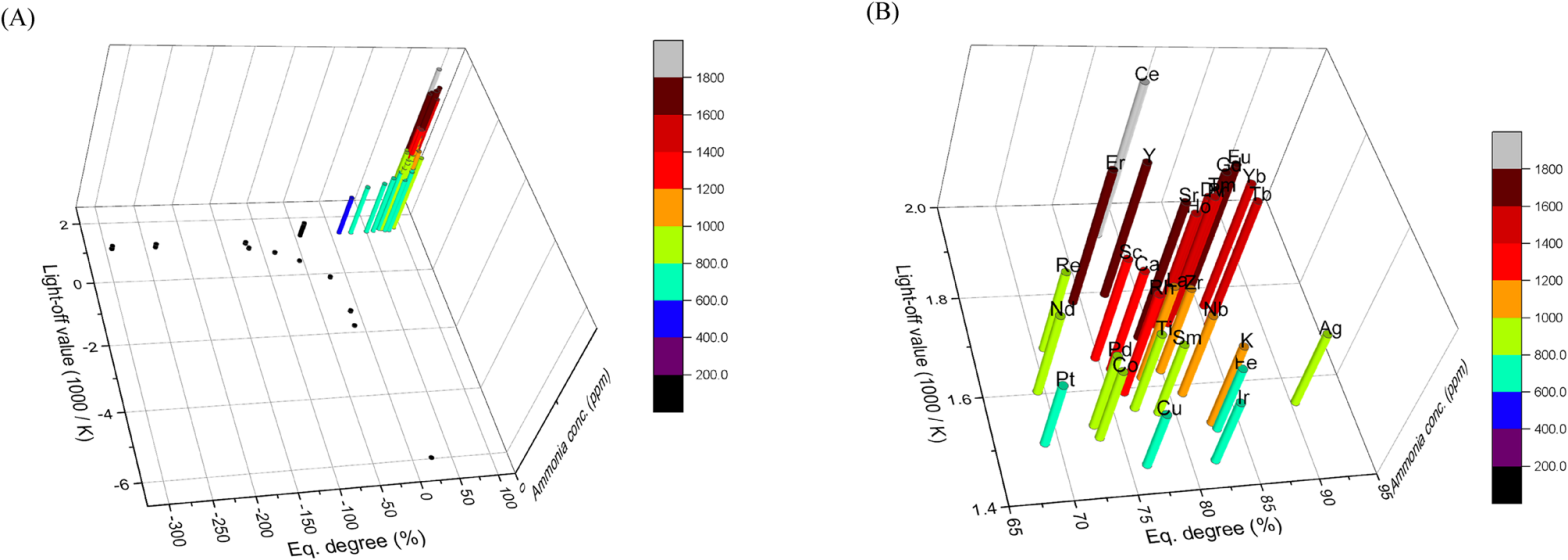
Correlation between Maximum ammonia concentration, equilibrium achievement degree and light-off value. (**A**) All catalysts. (**B**) Enlarged view of the crowded region in panel (**A**). The elemental symbols correspond to the added elements.

Figure 6 shows the relationships between the equilibrium achievement degree (X-axis), light-off value (Y-axis), and maximum ammonia concentration (Z-axis). We believe that such a graph, which includes all three of these evaluation axes, is important for evaluating catalysts for renewable energy applications. The three axes include: the light-off value, which reveals low-temperature high activity, the equilibrium achievement degree, which reveals the influence of the reverse reaction, and the maximum ammonia concentration, which simply reveals catalysts that exhibit high catalytic activities. The light-off value axis is the most important of these three because it reveals which catalysts can use hydrogen at low temperature and identifies catalysts that are robust and can be used in a catalytic reactor that may start up and shut down frequently. The next important factor is the maximum ammonia concentration or the equilibrium achievement degree. The maximum ammonia concentration exhibited a linear relationship with the light-off value in the present study; consequently, no special trend was observed. However, a catalyst with low catalytic activity is simply a difficult catalyst to use. Equilibrium achievement degree is a difficult indicator to rank. A catalyst with a low value may be strongly affected by the reverse reaction. However, in order to determine the reason for the low values, other methods of catalytic chemistry must be used. Since the indicators are lumped together for simple screening, it is better to interpret them as indicators of trend analysis. In addition, the reduction of the effect of reverse reactions and reaction conditions approaching equilibrium can be approached from process development rather than from catalyst development. It is important to understand that the equilibrium achievement is an index that encompasses various phenomena in the high temperature region. In conclusion, the catalyst with the highest height and located as far as possible to the upper right in the graph of the three axes is the catalyst that is suitable for the use of renewable energy.

## Conclusion

In this study, we investigated a simple and intuitive method of evaluating catalysts for the use of hydrogen derived from renewable energy. The light-off-value parameter adopted in this study are used in existing methods for evaluating catalysts; however, they are used in fields other than renewable energy. We consider this evaluation method to be suitable given the fluctuating characteristics of renewable energy. In other words, the method used to evaluate automotive exhaust gas catalysts is possibly more suitable for evaluating catalysts for use with renewable energy because automotive catalysts are evaluated under highly variable conditions. The equilibrium achievement, which comprehensively shows the effect of the reverse reaction, would be very useful when there is a difference between the thermodynamic theoretical value and the kinetic interpretation in the high temperature regime. The three-axis evaluation method developed in this study, which combines the above evaluation methods with catalytic activity data, is considered the most intuitive and suitable for rapid screening without the need for quantitative discussions or detailed catalyst analysis. From a different point of view, it may be good to evaluate light-off performance in the low temperature range and maximum ammonia concentration in the high temperature range, and to separate the temperature range.

However, it is also true that this evaluation method has shortcomings in that it seeks simplicity and intuitiveness, making it difficult to discuss quantitatively and in detail. Other evaluation methods, such as dynamic tracking and durability under fluctuating conditions, which were not included as catalyst-evaluation axes in this study, are further candidate methods for evaluating catalysts. Given that efficient use of renewable energy is becoming increasingly required, current methods for evaluating catalysts need to be reviewed.

## Methods

### Catalyst preparation

All chemicals were purchased from TANAKA KIKINZOKU KOGYO K.K., N.E. CHEMCAT, FUJIFILM Wako Pure Chemical Corporation, Furuya Metal Co., Ltd., or Strem Chemicals, Inc., and were used as received. Mg(NO_3_)_2_·6H_2_O and the metal nitrate (Mg:M = 5:1) were dissolved in water to approximately 20-times their combined weight. The pH of the aqueous solutions was adjusted to 10 using aqueous ammonia (approximately twice as many moles of NH_3_ as Mg + M) and the pH-adjusted solutions were left overnight. The precipitate from each solution was collected by filtration (twice) using filter paper. The MgO-MO_x_ supports were obtained by calcining the residues at 600 °C for 4 h in a muffle furnace. The apparent densities and true densities of the MgO-MO_x_ supports were measured to determine the void volumes of the MgO-MO_x_ supports. Ru(NO_3_)_3_ or [Ru(OH)(NO)(NH_3_)_4_](NO_3_)_2_ was used as the Ru precursor, which was diluted with water such that the MgO-MO_x_ support was immersed in twice as much Ru solution as the void volume of the support (the concentration of Ru was adjusted to 1 wt% loading). Ru was also supported by the impregnation method. The catalysts were calcined in a tubular furnace under a stream of 10% H_2_/N_2_ at 300 °C for 1 h. The calcined catalysts were sieved to particles 250-μm in size.

### Catalytic reactions

Catalytic activity was measured using a fixed-bed flow reactor^28^. A portion of the catalyst (0.2 g) was placed on quartz glass wool in the middle of the reactor, and a thermocouple was set up in the catalyst bed. The sample was pretreated under a stream of H_2_ (60 mL/min) and N_2_ (20 mL/min) gas at 600 °C for 30 min. Ammonia production was measured at under atmospheric pressure in the 250–600 °C temperature range under a stream of H_2_ (60 mL/min) and N_2_ (20 mL/min) gas, as detailed below. Steady-state amounts of generated ammonia were measured 90 min after each measurement temperature was reached. The product gases were analyzed by Fourier-transform infrared (FT-IR) spectroscopy (Nicolet iS50 FT-IR spectrophotometer, Thermo Fisher Scientific) using a multi-reflection gas cell (optical path length: 2.4 m, wavelength range: 4000–600 cm^−1^, resolution: 0.5 cm^−1^). The NH_3_ concentration was quantified using a calibration curve prepared using gas standards. The FT-IR absorbance band at 1122 cm^−1^ corresponds to NH_3_.

## References

[1] Kojima H, Matsumoto H, Tsujimura T (2017). Development of large scale unified system for hydrogen energy carrier production and utilization: Experimental analysis and systems modeling. Int. J. Hydrog. Energy..

[2] Osman, A. I., Mehta, N., Elgarahy, A., M., Hefny, M., Al-Hinai, A., Al-Muhtaseb., A. H. & Rooney, D. W., Hydrogen production, storage, utilisation and environmental impacts: A review. *Environ*. *Chem*. *Lett*., **20**, 153 (2022).

[3] Niermann M, Beckendorff A, Kaltschmitt M, Bonhoff K (2019). Liquid Organic Hydrogen Carrier (LOHC)—Assessment based on chemical and economic properties. Int. J. Hydrog. Energy..

[4] Yadav M, Xu Q (2012). Liquid-phase chemical hydrogen storage materials. Energy Environ. Sci..

[5] Rollinson AN, Jones J, Dupont V, Twigg MV (2011). Urea as a hydrogen carrier: a perspective on its potential for safe, sustainable and long-term energy supply. Energy Environ. Sci..

[6] Hamilton CW, Baker RT, Staubitz A, Manners I (2009). B-N compounds for chemical hydrogen storage. Chem. Soc. Rev..

[7] Makepeace, J. W., He, T., Weidenthaler, C., Jensen., T. R. Chang, F., Vegge, T., Ngene, P., Kojima, Y., Jongh., P., E., Chen, P. & David, W. I.F. Reversible ammonia-based and liquid organic hydrogen carriers for high-density hydrogen storage: Recent progress. *Int*. *J*. *Hydrog*. *Energy*. 44, 7746 (2019).

[8] Schüth F, Palkovits R, Schlögl R, Su DS (2012). Ammonia as a possible element in an energy infrastructure: catalysts for ammonia decomposition. Energy Environ. Sci..

[9] Okafor, E. G., Kurata, O., Yamashita, H., Inoue, T., Tsujimura, T., Iki, N., Hayakawa, A., Ito, S., Uchida, M. & Kobayashi, H. Liquid ammonia spray combustion in two-stage micro gas turbine combustors at 0.25 MPa; Relevance of combustion enhancement to flame stability and NOx control. *Appl. Energy Combust. Sci.* **7**, 100038 (2021).

[10] Juangsa FB, Irhamna AR, Aziz M (2021). Production of ammonia as potential hydrogen carrier: Review on thermochemical and electrochemical processes. Int. J. Hydrog. Energy..

[11] Lan R, Irvine JTS, Tao S (2012). Ammonia and related chemicals as potential indirect hydrogen storage materials. Int. J. Hydrogen Energy.

[12] Nanba T, Nagata Y, Kobayashi K, Javaid R, Atsumi R, Nishi M, Mochizuki T, Manaka Y, Kojima H, Tsujimura T, Matsumoto H, Fujimoto T, Suzuki K, Oouchi T, Kameda S, Hoshino Y, Fujimoto S, Kai M, Fujimura Y (2021). Explorative study of a Ru/CeO2 catalyst for NH3 synthesis from renewable hydrogen and demonstration of NH3 synthesis under a range of reaction conditions. J. Jpn. Petro. Inst..

[13] Ogura Y, Sato K, Miyahara S, Kawano Y, Toriyama T, Yamamoto T, Matsumura S, Hosokawa S, Nagaoka K (2018). Chem. Sci..

[14] Kitano M, Kujirai J, Ogasawara K, Matsuishi S, Tada T, Abe H, Niwa Y, Hosono H (2019). Low-temperature synthesis of perovskite oxynitride-hydrides as ammonia synthesis catalysts. J. Am. Chem. Soc..

[15] Hattori M, Iijima S, Nakao T, Hosono H, Hara M (2020). Nat. Commun..

[16] Kitano M, Inoue Y, Sasase M, Kishida K, Kobayashi Y, Nishiyama K, Tada T, Kawamura S, Yokoyama T, Hara M, Hosono H (2018). Angew. Chem. Int. Ed..

[17] Aika, K., Ohya, A., Ozaki, A., Inoue, Y. & Yasumori, I. Support and promoter effect of ruthenium catalyst: II. Ruthenium/alkaline earth catalyst for activation of dinitrogen, J. Catal., **92,** 305 (1985).

[18] Javaid R, Aoki Y, Nanba T (2020). Highly efficient Ru/MgO–Er2O3 catalysts for ammonia synthesis. J. Phys. Chem. Solids.

[19] Haneda M, Nakamura Y, Yamada T, Minami S, Kato N, Iwashina K, Endo Y, Nakahara Y, Iwachido K (2021). Comprehensive study of the light-off performance and surface properties of engine-aged Pd-based three-way catalysts. Catal. Sci. Technol..

[20] Nanba T, Chino T, Masukawa S, Uchisawa J, Obuchi A (2013). Total oxidation of toluene over Cu/TiO2/SiO2. Bull. Chem. Soc. Jpn..

[21] Olivier P, Bourasseau C, Bouamama PB (2017). Low-temperature electrolysis system modelling: A review. Renew. Sustain. Energy Rev..

[22] Niwa Y, Aika K (1996). The effect of lanthanide oxides as a support for ruthenium catalysts in ammonia synthesis. J. Catal..

[23] Aika K (2017). Role of alkali promoter in ammonia synthesis over ruthenium catalysts—Effect on reaction mechanism. Catal. Today.

[24] Rahat J, Matsumoto H, Nanba T (2019). Influence of reaction conditions and promoting role of ammonia produced at higher temperature conditions in its synthesis process over Cs-Ru/MgO catalyst. ChemistrySelect.

[25] Zhang J, Xu H, Ge Q, Li W (2006). Highly efficient Ru/MgO catalysts for NH3 decomposition: Synthesis, characterization and promoter effect. Catal. Commun..

[26] Ju, X., Liu, L., Zhang, X., Feng, J., He, T. & Chen. P. Highly efficient RU/MGO catalyst with surface-enriched basic sites for production of hydrogen from ammonia decomposition. ChemCatChem, **11**, 4161 (2019).

[27] Fujitani T, Nakamura I, Hashiguchi Y, Kanazawa S, Takahashi A (2020). Effect of catalyst preparation method on ammonia decomposition reaction over Ru/MgO catalyst. Bull. Chem. Soc. Jpn..

[28] Manaka Y, Nagata Y, Kobayashi K, Kobayashi D, Nanba T (2020). The effect of a ruthenium precursor on the low-temperature ammonia synthesis activity over Ru/CeO2. Dalton Trans..

